# Traversing the Highwire from Pop to Optical

**DOI:** 10.1371/journal.pbio.0030136

**Published:** 2005-04-12

**Authors:** Christopher W Tyler

## Abstract

A visual neuroscientist comments on the art of Roy Lichtenstein, as viewed in a recent exhibition at the San Francisco Museum of Modern Art

**Figure:**
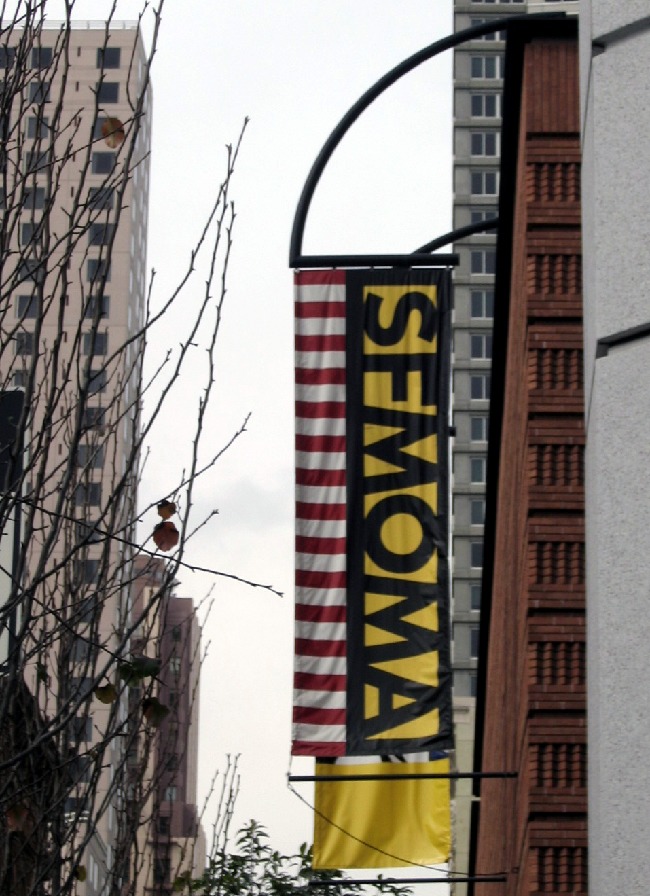


On the basis of his retrospective at the San Francisco Museum of Modern Art, it is evident that Roy Lichtenstein forged a narrow trajectory through the thickets of contemporary art. Unlike some of his protean fellow artists, such as David Hockney or Frank Stella, he found his style early in his career and followed its course with almost scientific precision for his entire life. Through an obsessive focus on the techniques of graphic production, he turned popular imagery into a high form of art and an exploration of the limits of visual perception.

The core concept of Lichtenstein's art is the iconic—drastic simplification of line and shadowing to reduce to the Platonic ideal of the object depicted—golf ball, truck tire, composition book, girl in love, or even the basic brushstroke of a daub of paint [1]. The basic inspiration of these icons is advertising imagery and comic book frames. Throughout his artistic life, he drew his imagery from the detritus of printed material—gum wrapper comics and small ads from the backs of newspapers—deliberately elevating visual sources at the opposite pole from the high art of the Renaissance. His first comic works were motivated by his sons, who challenged him by saying if he was such a great artist, could he draw Mickey Mouse for them? Lichtenstein was so stimulated by the result that he worked in the comic book style from then on. Interesting, Chuck Close tells a similar story about his own work, although in reverse. In his case a group of school kids were not “getting” his art and challenged him in the same way to prove his expertise. Close passed the test, but continued in his compulsive style, exploring the minutiae of the pixel structure in his grandiose portraits, while Lichtenstein explored the iconographic largesse of the new domain opened up by this challenge.

Lichtenstein's cartoon style of representation can be seen as an ironic commentary on the elitism of art, implying that art is merely a selection from the endless variety of images that bombard us. His self-mockery is exemplified by the second picture in the exhibition, a six-foot canvas bearing the letters “ART” [2]. In this piece Lichtenstein is explicitly interrogating the artistic community on the boundaries of the concept of art. Is the raw symbol placed in a gallery sufficient to constitute an artistic statement? This was a game that artists had been playing in earnest since the turn of the 20th century—Lichtenstein came up with one more variation on the theme. His later work consists of an extensive series of graphic “reflections” on the themes of classical art, from the second period of Pompeii, to the light explorations of the Impressionists, to Chinese landscapes, all in his egregious comic book pastiche.

The iconic is by now commonplace—the idea that cartoons capture the essence of an image was popularized by Fred Attneave in 1954 [3], when he drew attention to the way the outline of an object (and, indeed, the points of maximum curvature of the outline) captures most of the information of the full object image. The rest of the image can be thrown away without significant loss of its import. Nobel-Prize-winning experimental work conducted by David Hubel and Torsten Wiesel in the late 1950s consolidated this notion with the discovery that individual neurons in the visual cortex can be characterized as simple line detectors, i.e., that they are most active when a line of a particular orientation is found in a particular part of the visual world [4]. The work of Hubel and Wiesel illustrated that neural processing encodes the most relevant features in complex images. Indeed, they discovered a population of neurons called end-stopped cells that respond to the ends of lines and points of high line curvature in the manner required by Attneave's analysis. Interestingly, this confluence immediately predated Lichtenstein's entrée into the world of the comic book image, but the relationship seems to be coincidental in view of the iconographic symbolism of his choices. The likelihood that he had heard of these scientific developments seems remote, particularly in view of the fact that Andy Warhol was following the same track of using cartoon material, reputedly in mutual ignorance of Lichtenstein's breakthrough.

Beyond the iconographic, Lichtenstein plays with the visual impression derived from enlarging the halftone dots of the gum-wrapper comics. Through time, the dots become progressively larger and more insistent, emerging from their role as background fillers to dominate the entire canvas in a dizzying field of scintillations. In this sense, Lichtenstein seems to go beyond the role of cultural expositor and gadfly to explore the sensory implications of the optical redundancy of the printer's screen. The regular dot arrays shimmer and scintillate, ingraining themselves in our neural memory and projecting onto the gallery walls and neighboring paintings in a reminder of our visual fallibility. Such effects represent a resonance with so-called optical art (“op art”), a style promoted in the mid 1960s—by Bridget Riley and Victor Vasarely, in particular—that relies on visual illusion generated at the early levels of the nervous system: the retinal, the receptoral, the oculomotor, and the neural. Despite its name, it is not concerned with strictly optical effects such as diffraction, diffusion, interference, scintillation, polarization, and related optical phenomena. It is concerned with the visual and perceptual effects of dancing grids, jazzy dots, clashing colors, sliding waves, and so on.

Many of Lichtenstein's effects are a by-product of the printer's screen structure that is enlarged along with the other details of the printed image structure. His work seems to have been a major precursor of the op art movement, although he is not generally identified as a member of it. Indeed, he plays with the dot-screen as a theme in his later works, notably in the vast *Mirror in Six Panels* (1971), which shows nothing but the mirror surface reflecting empty space, apparently rendered in the transparent sheets of Benday dots in common use by graphic artists. Refreshingly, this is one of the few works that does not contain references to other art genres, but jousts with the concept of the image itself, again a reflection of nothing at all.

For the visual scientist, the most compelling painting in the exhibition may be *Rouen Cathedral Set V* (1969) [5], a meditation on Claude Monet's mediation on Rouen Cathedral, itself a series of impressionistic paintings of the cathedral in different lighting conditions. Here Lichtenstein abandons the cartoon-style bravura of line and text bubbles in a triplet of silk-screen close-ups of Monet's painterly impressions, differing only in the choice of colors for the three panels. The dot-screen now plays the role of a muslin or gauze curtain through which the cathedral is glimpsed, forming a vibrant haze that formalizes the image space into a kind of crystallized transparency that never quite settles into known categories of visual experience. While the left and right panels of the triptych are in bold shades of color, the central panel is rendered in accurate red-green isoluminance. As discovered by Richard Gregory, form processing is much weakened when the luminance differences are removed and forms are represented in colors that are accurately equated for their luminance values [6]. Although the colors are well seen, the form seems to shimmer and fluctuate, indicating that the shape-processing mechanisms are not well activated by the pure color differences. In Lichtenstein's Monet, the shimmer of the isoluminance interplays with the shimmer of the dot-screen to evoke a visual enigma, as we explore the image space to see whether the structure is indeed the same as in the flanking panels. One of the pleasures of art is its ability to slow down our sensory processing so that we become aware of the processes themselves, not just their symbolic role in our goal-oriented lives. Very few artists have played with the power of isoluminance to achieve this role in form processing: Lichtenstein seems to have been on to this property a decade earlier than Gregory, although he soon retreats back to the boldness of his cartoon pop-art style to explore a potpourri of the icons of classic sources.

There is much more that could be analyzed to place Lichtenstein in an art-historical framework, but perhaps one should just enjoy the power of the concrete image, simplified to its high-tone essentials and projected at large visual angle onto our excitable retinas. One comes away from the exhibit with a sense of the power of raw imagery that one may not have felt since the grade-school days of reading illicit comics when one was supposed to be learning the dates of battle sequences through history. But what does Lichtenstein's dot obsession reveal about neural processing? Why does repeated fine-grain structure wreak such havoc with our visual stability? This question was raised, in particular, by Donald MacKay with his high-density radial ray figure (Figure 1), which generates powerful complementary effects in both current viewing and as an aftereffect [7]. Just why high-density dots and lines elicit such powerful responses from our visual apparatus remains unexplained. Indeed, the issue does not seem even to be a topic of current research interest, despite the proliferation of research activities in visual processing in general. Contemplation of an exhibit such as Lichtenstein's sparks a realization of the wealth of neural processes still to be studied and explored.

**Figure 1:**
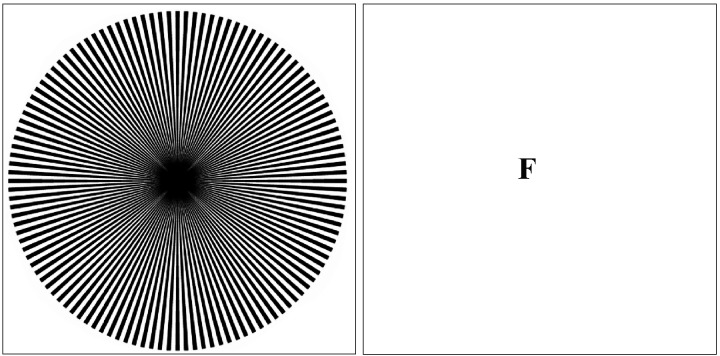
MacKay Ray Figure Notice the chrysanthemum effect while viewing the image. Fixate for ten seconds on the center of the figure, then transfer gaze to the blank fixation (F) and notice the streaming circular effects in the blank area, roughly orthogonal to the orientation of the rays.
